# Triple MAPK inhibition salvaged a relapsed post-BCMA CAR-T cell therapy multiple myeloma patient with a BRAF V600E subclonal mutation

**DOI:** 10.1186/s13045-022-01330-3

**Published:** 2022-08-17

**Authors:** Muhammad Elnaggar, Sarita Agte, Paula Restrepo, Meghana Ram, David Melnekoff, Christos Adamopoulos, Mark M. Stevens, Katerina Kappes, Violetta Leshchenko, Daniel Verina, Sundar Jagannath, Poulikos I. Poulikakos, Samir Parekh, Alessandro Laganà

**Affiliations:** 1 Tisch Cancer Institute, Icahn School of Medicine at Mount Sinai, New York, NY USA; 2 Department of Oncological Sciences, Icahn School of Medicine at Mount Sinai, New York, NY USA; 3 Graduate School of Biomedical Sciences, Icahn School of Medicine at Mount Sinai, New York, NY USA; 4 Travera, Medford, MA USA; 5 Department of Genetics and Genomic Sciences, Icahn School of Medicine at Mount Sinai, New York, NY USA

**Keywords:** BCMA CAR-T relapse, BRAF (V600E), MAPK inhibition, Clonality, RNA-seq, Whole-exome sequencing, Multiple myeloma

## Abstract

**Background:**

Multiple Myeloma (MM) is a progressive plasma cell neoplasm characterized by heterogeneous clonal expansion. Despite promising response rates achieved with anti-BCMA CAR-T cell therapy, patients may still relapse and there are currently no clear therapeutic options in post-CAR-T settings. In this report, we present a case of a post-BCMA CAR-T relapsed/refractory (RR) MM patient with skin extramedullary disease (EMD) in which a novel MAPK inhibition combinatorial strategy was implemented based on next-generation sequencing and in vitro experiments.

**Case presentation:**

A 61-year-old male with penta-refractory MM penta- (IgA lambda), ISS stage 3 with hyperdiploidy, gain of 1q21 and del13 was treated with anti-BCMA CAR-T cell therapy, achieving a best response of VGPR. He progressed after 6 months and was salvaged for a short period with autologous stem cell transplantation. Eventually, he progressed with extramedullary disease manifested as subcutaneous nodules. Based on whole-exome sequencing, we identified a BRAF (V600E) dominant subclone in both bone marrow and cutaneous plasmacytoma. Following in vitro experiments, and according to our previous studies, we implemented a triple MAPK inhibition strategy under which the patient achieved a very good partial response for 110 days, which allowed to bridge him to subsequent clinical trials and eventually achieve a stringent complete response (sCR).

**Conclusion:**

Here, we show the applicability, effectiveness, and tolerability the triple MAPK inhibition strategy in the context of post-BCMA CAR-T failure in specific subset of patients. The triple therapy could bridge our hospice bound RRMM patient with BRAF (V600E) to further therapeutic options where sCR was achieved. We will further evaluate triple MAPK inhibition in patients with BRAF V600E in a precision medicine clinical trial launching soon.

**Supplementary Information:**

The online version contains supplementary material available at 10.1186/s13045-022-01330-3.

## Background

Relapsed/Refractory Multiple Myeloma (RRMM) patients with extramedullary disease (EMD) have limited response to existing strategies like immunomodulatory drugs, proteasome inhibitors, and monoclonal antibodies. While anti-BCMA CAR-T has achieved prominent response in RRMM patients, post-CAR-T relapse represents a challenging therapeutic course with poor prognosis. About 53% of RRMM patients have shown emerging clones harboring mutations within the MAPK signaling pathway, including the targetable BRAF(V600E) in 7% of cases[1]. Targeting the MAPK signaling pathway with BRAF monomer selective inhibitors ± MEK inhibitor has been frequently attempted with RRMM patients with equivocal outcome on both disease progression and survival. This could be ascribed to feedback recovery of the MAPK pathway via BRAF dimers induction. Our prior studies have revealed that the multi-kinase inhibitor regorafenib is a potent inhibitor of dimeric BRAF. Moreover, we have demonstrated that negative feedback can be overcome by inhibiting BRAF in both its monomeric and dimeric form in combination with MEK inhibition leading to more efficacious and tolerable treatment in preclinical BRAF (V600E) cancer models. Patients with BRAF V600E often benefit from targeted therapy using small molecules inhibitors of monomeric BRAF V600E in combination with MEK inhibition [2]. This strategy, however, may fail due to feedback activation of the pathway via induction of BRAF dimer formation [3, 4]. Our recent study has shown efficacious pathway inhibition when the multi-kinase inhibitor regorafenib was added to the standard strategy. Regorafenib could potently inhibit dimeric BRAF and relieve the induced negative feedback [5]. In this report, we describe the case of an RRMM patient who was successfully treated with triple MAPK inhibition following relapse from CAR-T therapy.

## Case presentation

**Fig. 1:**
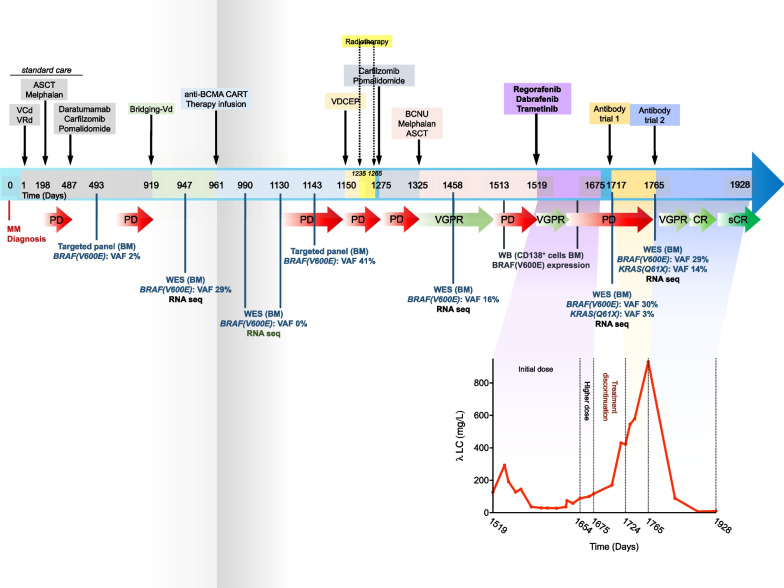
Timeline depicting the patient’s clonal evolution, treatment regimens, and responses since diagnosis. Timeline is represented in days since the establishment of MM diagnosis. Treatment regimens are indicated with black arrows descending toward the upper side of the timeline. Diagnostic whole-exome sequencing (WES), RNA-sequencing (RNA-seq) or targeted panel are indicated as blue bars descending from the timeline. Red arrows indicate period of disease progression (PD), and different shades of green arrows indicate very good partial response (VGPR), complete response (CR), and stringent complete response (sCR). Lower part of the graph depicts the patient’s lambda LC levels (mg/L) since the beginning of the triple therapy regimen (regorafenib, dabrafenib and encorafenib) until sCR achieved after antibody trial 2. ASCT: Autologous stem cell transplantation; VCd: Velcade (Bortezomib), cyclophosphamide and dexamethasone. VRd: Velcade (Bortezomib), lenalidomide, and dexamethasone; Vd: Velcade (Bortezomib) and dexamethasone; anti-BCMA CAR-T: anti-B cell maturation antigen chimeric antigen receptor T cells; VDCEP: Velcade (Bortezomib), dexamethasone, cyclophosphamide, etoposide, and cisplatin; BCNU: Carmustine

A 61-year-old male with penta-refractory MM (IgA lambda), ISS stage 3 with hyperdiploidy, gain of 1q21 and del13 was treated with anti-BCMA CAR-T cell therapy, achieving a best response of VGPR. He progressed after 6 months and was temporarily salvaged with BCNU/Melphalan (carmustine 300 mg/m2 and melphalan 140 mg/m2) as conditioning regimen before ASCT, achieving a best response of PR until further progression with extramedullary disease (subcutaneous skin lesions in lower extremities) and elevated lambda free light chains (FLC, 126.4 mg/l) at 6 months (Fig. 1) (Additional file 1).

**Fig. 2:**
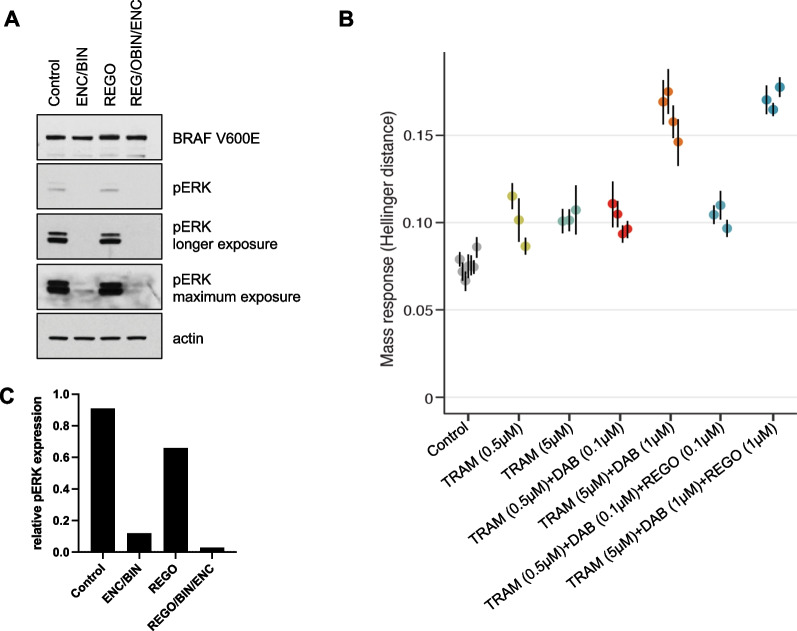
Triple MAPK inhibition effectively reduces phosphorylated ERK in BRAF (V600E) CD138^+^ plasma cells **A** WB of magnetic bead selected CD138^+^ plasma cells from RRMM patient’s BMA after 48 h in vitro treatment with encorafenib (ENC; 50 nM) and binimetinib (BIN; 250 nM), regorafenib alone (REG; 1μΜ), or combination of the three drugs. **B** Travera analysis on RRMM patient CD138 + cells showing sensitivity to trametinib (TRAM) in combination with dabrafenib (DAB) and regorafinib (REG) at varying concentrations. **C** Relative pERK protein expression after quantification and normalization to actin

Prior to CAR-T therapy, targeted and WES had identified a BRAF(V600E) mutation in his BM, which persisted throughout treatment and was also detected in a cutaneous plasmacytoma with a variant allele frequency (VAF) of 41% at relapse from CAR-T. Western blot (WB) analysis of the post-CAR-T relapse bone marrow aspirate (BMA) confirmed the BRAF(V600E) mutation and further showed phosphorylation of ERK (pERK), consistent with activation of MAPK signaling, which was inhibited by combining the MEK inhibitor binimetinib with the BRAF(V600E) monomer inhibitor encorafenib. Imaging the blot again with longer exposure time revealed small residual MAPK activity, which was abrogated by adding the BRAF dimer inhibitor regorafenib (Fig. 2A and C). Further assessment of ex vivo drug sensitivity of the patient’s CD138^+^ MM cells post-CAR-T to different combinations of MAPK inhibitors through a functional assay based on measurements of single-cell mass response suggested high efficacy of the combination of trametinib at 5 µM and dabrafenib at 1 µM. Adding regorafenib at 1 µM indicated improved response compared to the double combination (Fig. 2B) [6]. According to our in vitro experimental results and previous literature on MAPK pathway homeostasis, phosphorylated ERK—the downstream effector of activated MAPK pathway—exerts a negative feedback inhibition on the receptor tyrosine Kinase (RTK). When pERK levels decrease upon RAF monomer inhibition ± MEK inhibition (standard approach), this negative feedback is eliminated, i.e., Relieve of negative feedback, resulting in RTK and subsequent MAPK activation, which is known as feedback recovery. This is mostly mediated by the formation of dimeric RAF. Therefore, adding a dimer selective RAF inhibitor, i.e., regorafenib, overcomes this feedback recovery [7–9].

Based on these findings and assessment, the patient was started on targeted therapy with a combination of a BRAF monomer inhibitor, dabrafenib (100 mg, orally twice daily), a MEK inhibitor, trametinib (1.5 mg, orally for 21/28 days daily), and a BRAF dimer inhibitor, regorafenib (40 mg, orally once daily). Within 3 months of treatment initiation, prompt reduction in subcutaneous skin lesions and 80% reduction in Lambda free light chain (λFLC) (27.5 mg/l) were observed (Fig. 1). Furthermore, the patient had good tolerance to all three medications with minimal side effects (grade 1 fatigue) which allowed him to carry out activities of daily living and return to work.

After more than three months of optimal treatment response, the patient’s FLC started to gradually increase, indicating reduced response to the triple MAPK inhibition, albeit with no recurrence of the subcutaneous nodules. The triple MAPK inhibition dose was maximized to dabrafenib 150 mg (orally twice daily), trametinib 2 mg (orally once daily), and regorafenib 80 mg (orally once daily), for less than a month, and eventually discontinued when no further response was observed with FLC peaking at 116.6 mg/l (Fig. 1). The 110-day response period achieved by the triple MAPK inhibition allowed the patient to become eligible for clinical trials and is currently benefiting from bispecific treatment with a λFLC of 10.9 mg/l with FLC ratio of 0.49 indicating stringent complete response (sCR).

**Fig. 3:**
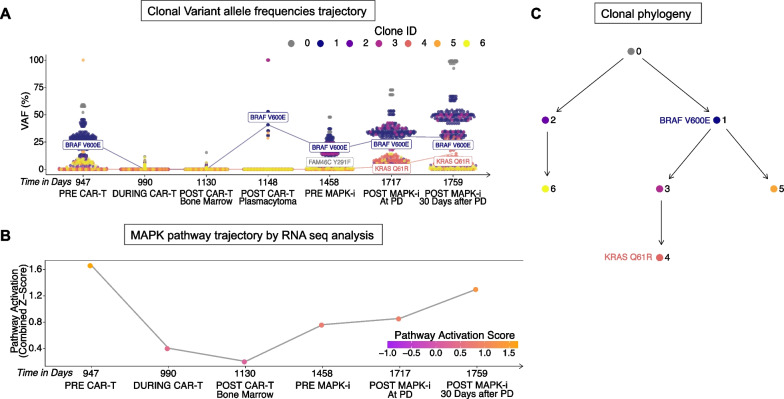
Temporal evolution and trajectory of MAPK alterations. **A** Clusters of subclonal mutations sampled over time with the mutational VAF represented as a percentage on the y axis show the trajectory of the subclone harboring the BRAF V600E mutation over time in response to treatment and the later emergence of a subclonal KRAS Q61R mutation after the end of triple MAPK inhibition. **B** A reconstructed phylogenetic tree of subclones across all time points shows that the BRAF V600E is subclonal and ancestral to the clone that gives rise to a KRAS Q61R mutation. **C** RNA expression shows an increase in MAPK pathway activation, measured as a combined z-score, over time in response to treatment with triple MAPK inhibition therapy

**Fig. 4:**
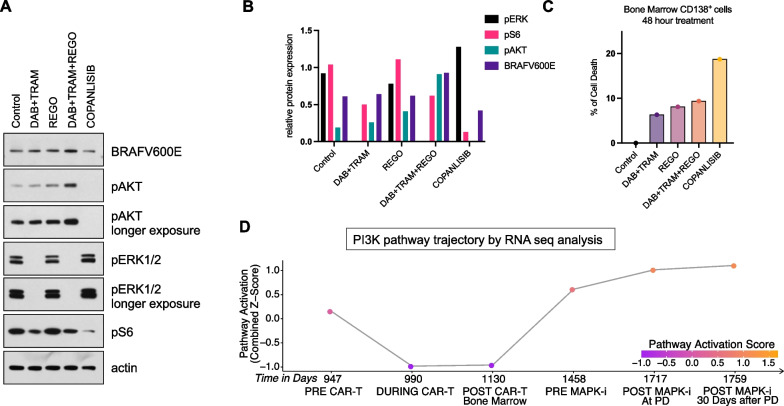
CD138^+^ BMA plasma cells shift their dependency to PI3K/AKT pathway with increased sensitivity to copanlisib **A** WB of magnetic bead selected CD138 + plasma cells from RRMM patient’s BMA after 48 h in vitro treatment with with encorafenib (ENC; 50 nM) and binimetinib (BIN; 250 nM), regorafenib alone (REG; 1μΜ), combination of the three drugs or copanlisib alone (25 nM). **B** Rlative protein expression of pERK, pS6, pAKT and BRAF (V600E) after quantification and normalization to actin **C** CD138 + viability measurement after 48 h in vitro treatment with with encorafenib (ENC; 50 nM) and binimetinib (BIN; 250 nM), regorafenib alone (REG; 1 μΜ), combination of the three drugs or copanlisib alone (25 nM). **D** RNA expression shows elevated PI3K/Akt pathway activation, measured as a combined z-score, throughout the course of treatment with triple MAPK inhibition therapy

To better understand the evolutionary trajectory of the alterations in the MAPK pathway over time, we analyzed matched DNA and RNA-sequencing from CD138^+^ samples taken at various time points from prior to CAR-T therapy to after relapse from triple MAPK inhibition. The BRAF V600E mutation was subclonal, yet detected relatively early within the tumor clonal phylogeny, and was present in the bone marrow prior to CAR-T therapy (VAF = 12.8%, Fig. 3A–B). During CAR-T therapy, the subclone harboring this mutation reduced in cellular fraction to undetectable levels, then recovered upon the patient’s relapse of CAR-T in the cutaneous plasmacytoma (VAF = 40.7%) and was finally detectable in the bone marrow prior to the start of triple MAPK inhibition (Fig. 3A). The BRAF V600E subclone further expanded at relapse from triple MAPK inhibition, producing a descendant subclone harboring a KRAS Q61R mutation (Fig. 3B). The new KRAS Q61R mutated subclone was detectable in trace proportions immediately after relapse from triple MAPK inhibition (VAF = 2.7%) and expanded further 30 days later (VAF = 14.4%, Fig. 3A). Longitudinal pathway analysis in RNA-seq data also revealed a successive upregulation of MAPK and PI3K pathway activity after relapse from CAR-T and throughout the course of MAPK inhibition (Figs. 3C and 4D).

Post-MAPK inhibition western blot analysis revealed only slight reduction in phosphorylated ribosomal S6 upon *ex vivo* triple MAPK therapy, despite effective inhibition of the MAPK pathway with undetectable level of phosphorylated ERK. This suggests that the patient’s plasma cell clones might have shifted their dependency to an alternative pathway, as suggested by increased PI3K signaling transcriptional output, based on RNAseq analysis. In fact, treatment of the MM cells with the PI3K inhibitor copanlisib could effectively inhibit phosphorylated ribosomal protein S6, indicating higher clonal dependency on PI3K (Fig. 4A and B). Pathway dependency was further evaluated by measuring cell death in a 48 h in vitro assay with patient’s selected CD138^+^ cells with different drug combination. Triple therapy achieved 10% cell death while copanlisib approximately doubled cell death of the plasma cell subclones (Fig. 4C). This indicates that eventual failure of triple MAPKi therapy may be a result of tumor clonal evolution, as well as a shift in cell dependency toward PI3K pathway activation.

## Discussion and conclusions

The clinical case presented in this report describes a MM patient who progressed on both ASCT and anti-BCMA CAR-T cell therapy and presented with high levels of FLC and subcutaneous nodules. Relapsing BCMA CAR-T usually represents a challenging treatment course due to limited therapeutic options. Post-CAR-T relapse WES of the patient’s BMA sample revealed persistence of a prior BRAF V600E mutation, which affects the MAPK pathway and is commonly present in solid tumors as well as 7% of RRMM [1].

In our patient, in vitro Post-CAR-T relapse western blot signaling analysis on BMA CD138^+^ suggested an advantage of triple MAPK inhibition compared to the standard strategy.

Triple MAPK inhibition was prescribed and orally administered to the patient. In three-month period, the patient has shown clinical improvement represented by 80% reduction in the subcutaneous nodules which also correlated to a drop of the FLC near to basal levels (27.5 mg/l). Moreover, the patient has shown tolerability to the administered regimen, as the addition of regorafenib allowed lower doses of both dabrafenib and trametinib, the latter being cardiotoxic at higher doses [10, 11].

After three months, the patient response to the triple therapy declined and was evident by gradual rise of the FLC but with no recurrence of the subcutaneous nodules. This indicates that the triple therapy could successfully eradicate the MM cell clones responsible for the cutaneous manifestation. WES analysis of the patient BMA at relapse from triple MAPK inhibition therapy has also shown a newly emerging clone harboring a KRAS Q61R mutation in addition to the previously existing BRAF V600E, which was associated with increased MAPK activity observed at the RNA level. This may explain the gradual decline of the patient response to the triple regimen.

Furthermore, RNA analysis showed increased PI3K pathway activation and, concordantly, BMA CD138^+^ signaling analysis post-triple MAPK inhibition by WB indicated that the patient might benefit from copanlisib, a PI3K inhibitor, either as a single agent or in combination with triple MAPK inhibition. Copanlisib could efficiently inhibit the phosphorylation levels of AKT and showed clones dependence on PI3K evident by marked drop of phosphorylation of ribosomal S6 protein compared to MAPK inhibition that showed only a marginal drop [12] [13].

In this case study, we have shown the applicability, effectiveness, and tolerability of the triple MAPK inhibition in hematological malignancy context to salvage post-BCMA CAR-T cell therapy in a RRMM patient with V600E mutation. This represents proof-of-concept that, while not curative, targeted molecules may serve as potential bridging therapies to clinical trial enrollment. We have also demonstrated the power of routine NGS and WB signaling pathway analysis in tracking clonal evolution and identifying targetable pathways which allow tailoring of personalized therapeutics for MM patients.

## Methods

### Next-generation sequencing

CD138^+^ selected cells were collected from BMA at multiple time points throughout the disease course from before CAR-T cell therapy to after relapse from triple MAPKi therapy. Genomic DNA was extracted from peripheral blood and sequenced as a matched normal control for variant calling. Additionally, a full BMA was collected from a patient’s cutaneous plasmacytoma sample at CAR-T cell therapy relapse for targeted sequencing via the FoundationOne Heme panel.

### Whole-exome sequencing

Raw reads were trimmed and assessed for quality control using fastp [14]. Trimmed reads were aligned with bwa mem [15], followed by processing via the Genome Analysis ToolKit (GATK) best practices pipeline [16]. Allele-specific copy number alterations were called using FACETS [17]. Somatic mutations were called using a consensus method to reduce false positives, and bam-readcount [18] was used to compute variant allele frequencies (VAFs). Variants called in at least two of three methods, Mutect2 [16], Lancet[19], and Strelka[20], with at least 10 reads supporting the alternate allele, were retained for further analysis. Pyclone-vi was used to cluster variants and their corresponding Copy Number Alterations (can) into subclonal populations across all sampled time points with the following parameters: 1000 grid points, 50 restarts, beta-binomial sampling density, and 20 maximum clusters [21]. Clone phylogenies were computed using a modified version of ClonEvol as implemented by the REVOLVER package in R with default parameters [22]. Downstream analysis, visualizations, and statistics were conducted using R.

### RNA-sequencing

Raw reads were trimmed and assessed for quality control using fastp [14], followed by alignment with STAR [23], and duplicate marking with Picard MarkDuplicates [16]. Gene-level counts were quantified using subread featureCounts [24]. The raw counts matrix was normalized with voom and batch corrected with the sva comBat package [25]. Pathway activation for the MAPK and PI3K/Akt pathways was assessed using the GSVA package in combined z-score mode [26]. The pathway definition for PI3K/Akt activation was defined by the following genes as described in the literature: *PI3KCA*, *IGF1*, *IGFR*, *AKT1*, *VEGF*, *VEGFR*, and *PDK1* [27]. The pathway definition for MAPK activation was defined by the following genes as previously described: *ETV1*, *ETV4*, *ETV5*, *FOS*, *FOSB*, *FRA1*, *MYC*, *DUSP4*, *DUSP6* [28].

### Western blot (WB)

Cells were washed with PBS and lysed on ice for 5 min in NP40 buffer (50 mmol/L Tris pH 7.5, 1% NP40, 150 mmol/L NaCl, 10% Glycerol 1 mmol/L EDTA) supplemented with protease and phosphatase inhibitors (Roche). Lysates were centrifuged at 15,000 rpm for 10 min, and the protein concentration was quantified using BCA (Pierce). Proteins were separated by NuPAGE and 4% to 12% Bis Tris Gel (Novex), and they were immunoblotted and transferred to nitrocellulose membranes (GE Healthcare) according to standard protocols. Membranes were immunoblotted overnight with antibodies against pERK1/2Thr202/Tyr204 (D13.14.4E), pAKTSer473 (D9E), pS6 Ribosomal Protein Ser240/244 (D68F8) and β-actin (13E5) from Cell Signaling; BRAFV600E from NewEast Biosciences. The next day, membranes were probed with anti-rabbit IgG or anti-mouse IgG secondary antibody (Cell Signaling), and chemiluminescent signals were detected on X-ray films.

### Mass response measurements

RRMM patient’s BMA selected CD138^+^ cells’ sensitivity to MAPK inhibitors were assessed by Travera. Selected cells are incubated with different inhibitors at different concentrations and combinations for 15 h before having their single-cell mass distributions measured. Inhibitor efficiency is then characterized by the relative statistical difference (Hellinger distance) between each test condition and untreated controls.

### Compounds

Encorafenib, trametinib, copanlisib, binimetinib, regorafenib were obtained from Selleckchem. Compounds were dissolved in DMSO to yield 10 mmol/L stock.

## Supplementary Information

**Additional file 1. **Laboratory measurement levels of light chain values.

## Data Availability

Raw sequencing data will be deposited in SRA upon publication.

## Abbreviations

MM: Multiple myeloma
EMD: Extramedullary disease
RR: Refractory/relapsed
MAPK: The mitogen activated protein kinase
ASCT: Autologous stem cell transplantation
PD: Disease progression
sCR: Stringent complete response
VGPR: Very good partial response
BCMA CAR-T: B cell maturation antigen chimeric antigen receptor T cells
FLC: Free light chain
BMA: Bone marrow aspirate
WES: Whole-exome sequencing
WB: Western blot
PI3K: Phosphatidylinositol-3 kinase
