# Correction: Triple MAPK inhibition salvaged a relapsed post-BCMA CAR-T cell therapy multiple myeloma patient with a BRAF V600E subclonal mutation

**DOI:** 10.1186/s13045-023-01449-x

**Published:** 2023-05-08

**Authors:** Muhammad Elnaggar, Sarita Agte, Paula Restrepo, Meghana Ram, David Melnekoff, Christos Adamopoulos, Mark M. Stevens, Katerina Kappes, Violetta Leshchenko, Daniel Verina, Sundar Jagannath, Poulikos I. Poulikakos, Samir Parekh, Alessandro Laganà

**Affiliations:** 1 Tisch Cancer Institute, Icahn School of Medicine at Mount Sinai, New York, NY USA; 2 Department of Oncological Sciences, Icahn School of Medicine at Mount Sinai, New York, NY USA; 3 Graduate School of Biomedical Sciences, Icahn School of Medicine at Mount Sinai, New York, NY USA; 4 Travera, Medford, MA USA; 5 Department of Genetics and Genomic Sciences, Icahn School of Medicine at Mount Sinai, New York, NY USA

**Correction : Journal of Hematology & Oncology (2022) 15:109** **https://doi.org/10.1186/s13045-022-01330-3**

The authors of the original article [1] mistakenly omitted a Funding source which is stated ahead: Alessandro Lagana was supported by the ASH Bridge Grant Award.
