# Supplementary material for: Triple MAPK inhibition salvaged a relapsed post-BCMA CAR-T cell therapy multiple myeloma patient with a BRAF V600E subclonal mutation
Source: J Hematol Oncol. 2022 Aug 17;15:109. doi: 10.1186/s13045-022-01330-3 (PMC9382834; doi:10.1186/s13045-022-01330-3)
Supplement: Supplementary file 1 — Additional file 1. Laboratory measurement levels of light chain values. [file 13045_2022_1330_MOESM1_ESM.docx]

**Supplementary Table:** Laboratory measurement levels of Kappa light chain (3.3 - 19.4 mg/l), Lambda light chain (5.7 - 26.3 mg/l), Kappa/Lambda ratio (KLR) (0.26 - 1.65) since the time of diagnosis and over the course of 56 months.

| **Test Order** | **Kappa** | **Lambda** | **KLR** |  |
| --- | --- | --- | --- | --- |
| 1st | 9.49 | 127.33 | 0.074531 |  |
| 2nd | 13.11 | 56.68 | 0.231299 |  |
| 3rd | 7.57 | 44.67 | 0.169465 |  |
| 4th | 9.21 | 29.04 | 0.317149 |  |
| 5th | 11.69 | 39.92 | 0.292836 |  |
| 6th | 14.61 | 40.82 | 0.357913 |  |
| 7th | 8.31 | 19.68 | 0.422256 |  |
| 8th | 10.53 | 21.63 | 0.486824 |  |
| 9th | 14.94 | 19.58 | 0.763023 |  |
| 10th | 17.01 | 24.75 | 0.687273 |  |
| 11th | 20.1 | 34.5 | 0.582609 |  |
| 12th | 12.8 | 499.6 | 0.02562 |  |
| 13th | 5.9 | 434.5 | 0.013579 |  |
| 14th | 1.8 | 15.2 | 0.118421 |  |
| 15th | 9.4 | 64.8 | 0.145062 |  |
| 16th | 1.7 | 192.8 | 0.008817 |  |
| 17th | 1.7 | 265.9 | 0.006393 |  |
| 18th | 1.9 | 107.7 | 0.017642 |  |
| 19th | 2.1 | 669.1 | 0.003139 |  |
| 20th | 2.2 | 304.4 | 0.007227 |  |
| 21st | 1.2 | 182.4 | 0.006579 |  |
| 22nd | 0.4 | 213.3 | 0.001875 |  |
| 23rd | 0.4 | 63.8 | 0.00627 |  |
| 24th | 0.4 | 6.2 | 0.064516 |  |
| 25th | 0.4 | 4.5 | 0.088889 |  |
| 26th | 0.7 | 6.1 | 0.114754 |  |
| 27th | 5.8 | 19 | 0.305263 |  |
| 28th | 8.9 | 203.7 | 0.043692 |  |
| 29th | 10 | 413.6 | 0.024178 |  |
| 30th | 9.7 | 471 | 0.020594 |  |
| 31st | 4.7 | 187 | 0.025134 |  |
| 32nd | 17 | 159.9 | 0.106316 |  |
| 33rd | 15.1 | 65.8 | 0.229483 |  |
| 34th | 0.5 | 678.3 | 0.000737 |  |
| 35th | 0.7 | 967 | 0.000724 |  |
| 36th | 0.7 | 192 | 0.003646 |  |
| 37th | 1.6 | 104.5 | 0.015311 |  |
| 38th | 7.8 | 39.4 | 0.19797 |  |
| 39th | 4.1 | 16.6 | 0.246988 |  |
| 40th | 7.2 | 65.9 | 0.109256 |  |
| 41st | 8 | 126.4 | 0.063291 | **Triple MAPK Inhibition (Initial Dose)** |
| 42nd | 7 | 292.8 | 0.023907 |  |
| 43rd | 6.4 | 191.9 | 0.033351 |  |
| 44th | 9.2 | 128 | 0.071875 |  |
| 45th | 7.3 | 145.1 | 0.05031 |  |
| 46th | 7.3 | 59 | 0.123729 |  |
| 47th | 6.6 | 36.6 | 0.180328 |  |
| 48th | 7.4 | 30.1 | 0.245847 |  |
| 49th | 9.3 | 29.4 | 0.316327 |  |
| 50th | 7.4 | 27.5 | 0.269091 |  |
| 51st | 9.1 | 33.1 | 0.274924 |  |
| 52nd | 7.6 | 36.3 | 0.209366 |  |
| 53rd | 8.7 | 58.3 | 0.149228 |  |
| 54th | 9.9 | 88.3 | 0.112118 | **Triple MAPK Inhibition (Maximum Dose)** |
| 55th | 10.2 | 100.6 | 0.101392 |  |
| 56th | 22 | 116.6 | 0.188679 | **Distcontinuation of Triple MAPK inhibition** |
| 57th | 12.9 | 96.4 | 0.133817 |  |
| 58th | 8.9 | 170.5 | 0.052199 |  |
| 59th | 7.8 | 276 | 0.028261 |  |
| 60th | 8.4 | 271.8 | 0.030905 | **Antibody Trial 1** |
| 61st | 8.8 | 368.4 | 0.023887 |  |
| 62nd | 7.8 | 394.4 | 0.019777 |  |
| 63rd | 7.2 | 523.6 | 0.013751 |  |
| 64th | 7.7 | 601 | 0.012812 |  |
| 65th | 7.6 | 485 | 0.01567 |  |
| 66th | 4.4 | 580.1 | 0.007585 | **Antibody Trial 2** |
| 67th | 0.9 | 703 | 0.00128 |  |
| 68th | 0.7 | 471.3 | 0.001485 |  |
| 69th | 0.4 | 224.5 | 0.001782 |  |
| 70th | 0.5 | 233.7 | 0.002139 |  |
| 71st | 1 | 66.4 | 0.01506 |  |
| 72nd | 5.8 | 11.8 | 0.491525 |  |
| 73rd | 2.4 | 7 | 0.342857 |  |
| 74th | 1.7 | 5 | 0.34 |  |
| 75th | 5.4 | 7.5 | 0.72 |  |
| 76th | 5.4 | 10.9 | 0.495413 |  |
| 77th | 1.2 | 5.1 | 0.235294 |  |
| 78th | 1.1 | 2.7 | 0.407407 |  |
| 79th | 5.1 | 5.7 | 0.894737 |  |
